# Recent Understanding of Soil Acidobacteria and Their Ecological Significance: A Critical Review

**DOI:** 10.3389/fmicb.2020.580024

**Published:** 2020-10-30

**Authors:** Sadaf Kalam, Anirban Basu, Iqbal Ahmad, R. Z. Sayyed, Hesham Ali El-Enshasy, Daniel Joe Dailin, Ni Luh Suriani

**Affiliations:** ^1^Department of Biochemistry, St. Ann’s College for Women, Hyderabad, India; ^2^Department of Plant Sciences, School of Life Sciences, University of Hyderabad, Hyderabad, India; ^3^Department of Agricultural Microbiology, Aligarh Muslim University, Aligarh, India; ^4^Department of Microbiology, PSGVP Mandal’s, Arts, Science and Commerce College, Shahada, India; ^5^Institute of Bioproduct Development, Universiti Teknologi Malaysia (UTM), Skudai, Malaysia; ^6^School of Chemical and Energy Engineering, Faculty of Engineering, Universiti Teknologi Malaysia (UTM), Skudai, Malaysia; ^7^City of Scientific Research and Technological Applications, New Borg El-Arab, Egypt; ^8^Biology Department, Faculty of Mathematics and Natural Science, Udayana University, Bali, Indonesia

**Keywords:** acidobacteria, biogeochemical cycles, ecological roles, metagenomics, molecular characterization, plant growth-promoting activities, soil

## Abstract

Acidobacteria represents an underrepresented soil bacterial phylum whose members are pervasive and copiously distributed across nearly all ecosystems. Acidobacterial sequences are abundant in soils and represent a significant fraction of soil microbial community. Being recalcitrant and difficult-to-cultivate under laboratory conditions, holistic, polyphasic approaches are required to study these refractive bacteria extensively. Acidobacteria possesses an inventory of genes involved in diverse metabolic pathways, as evidenced by their pan-genomic profiles. Because of their preponderance and ubiquity in the soil, speculations have been made regarding their dynamic roles in vital ecological processes *viz*., regulation of biogeochemical cycles, decomposition of biopolymers, exopolysaccharide secretion, and plant growth promotion. These bacteria are expected to have genes that might help in survival and competitive colonization in the rhizosphere, leading to the establishment of beneficial relationships with plants. Exploration of these genetic attributes and more in-depth insights into the belowground mechanics and dynamics would lead to a better understanding of the functions and ecological significance of this enigmatic phylum in the soil-plant environment. This review is an effort to provide a recent update into the diversity of genes in Acidobacteria useful for characterization, understanding ecological roles, and future biotechnological perspectives.

## Introduction

Prokaryotes, the unseen majority sustaining life on Earth, are involved in a multitude of interactions and biogeochemical processes having global ecological relevance, including decomposition, mineralization, storage, and release of nutrients (Sikorski, 2015). The number of prokaryotes present in a gram of soil can be between 10^6^ and 10^9^ cells (Bulgarelli et al., 2013). It is apparent from the above data that there is an abundant density of bacteria in the soil, however, the discrepancy is that only less than 1% bacteria from natural environments, including soil, could be cultivated by using conventional culturing techniques (Crits-Christoph et al., 2018; Chaudhary et al., 2019). A large segment of the microbial community gets consistently overlooked during routine microscopic analysis, indicating them to be novel and hitherto unrecognized with ambiguous physiology and growth requirements (Youssef et al., 2015). A rational investigation stemming from the recognition of this discrepancy is the identification of these refractive bacteria which escape conventional enrichment and isolation procedures. In this direction, the 16S rRNA gene, the gold standard for phylogenetic analysis, proves to be a useful tool for studying the evolution-based taxonomy of microbial communities from various econiches (Rosselli et al., 2016). The rare biosphere comprises of several identified novel bacterial lineages, which appear to be profoundly branching within the bacterial tree and remain unaffiliated with any known bacterial phyla and are termed as candidate phylum (or candidate division) (Solden et al., 2016).

Molecular studies in the soil revealed the presence of unseen, hidden, recalcitrant bacteria referred to as difficult-to-culture, hitherto-unculturable, or yet-to-be cultivated bacteria. In contrast to standard fast-growing bacteria, these underexplored bacterial groups remain elusive to conventional microbiological cultivation techniques, mostly because their optimized growth conditions are still underexplored. The proportion of such bacteria exceeds far more than that of the culturable ones. Among them, Acidobacteria constitutes the most abundant phylum whose members dominate soil bacterial communities (Vartoukian et al., 2010; Chaudhary et al., 2019). Data from 16S rRNA gene inventories signify the vastness and breadth of the phylum with genetic and metabolic diversity (Janssen, 2006). Study of these hitherto-unculturable constituents of soil microbial population through culture-dependent and -independent approaches revealed the essence of their bioactivity and their ecological functions in plant-soil ecosystems (Huber et al., 2016; Lladó et al., 2018; Elmagzob et al., 2019). The underexplored phylum Acidobacteria thus provides an enthralling and seamless source of biological diversity, offering new avenues for exploitation. Henceforth, the most recent advances mining the difficult-to-culture soil acidobacterial diversity for biotechnological advances and comprehensive understanding of the genetic diversity and ecophysiological profiles of Acidobacteria in the plant-soil econiche has been scrupulously presented in this review.

## Underexplored Phylum Acidobacteria

Acidobacteria represents an enigmatic phylum with its members copiously distributed in different ecosystems (Mushinski et al., 2018). Acidobacteria is ubiquitous in diverse terrestrial environments ranging from tundra soils to desert soils, from peatland soils and sediments to grasslands, forests, and agricultural lands (Janssen, 2006; Eichorst et al., 2018) and constitute about 5–70% of the soil microbial populace (Huber et al., 2016). The members are recalcitrant, rendering this phylum to be feebly understood (Hugenholtz et al., 1998; Huber et al., 2016). The recalcitrance of most Acidobacteria members to grow on conventional growth media can be attributed to their oligotrophic nature or ecological K-strategy (Ward et al., 2009; Kielak et al., 2016a). Thus, the use of low nutrient media, modified incubation conditions including elevated CO_2_, low pH, prolonged incubation periods, and supplementing growth media with amendments like antioxidants, rhizosphere extracts, etc., have led to the isolation of several acidobacterial species (Stevenson et al., 2004; Sait et al., 2006; da Rocha et al., 2009; Tanaka et al., 2017).

Based on the analysis of major 16S rRNA gene sequence clades, the phylum Acidobacteria is phylogenetically classified into 26 subdivisions (Barns et al., 2007). Among these, only seven subdivisions (namely the subdivisions 1, 3, 4, 6, 8, 10, and 23) are represented by taxonomically described members (Dedysh and Yilmaz, 2018; Eichorst et al., 2018). Although more than 12,000 distinct phylotypes and more than 6,500 species-level operational taxonomic units have been reported so far for this predominant soil bacterial group, yet it is described by only 56 cultivable species belonging to 28 genera (Overmann et al., 2017; Vieira et al., 2017; Dedysh and Yilmaz, 2018). All cultured Acidobacteria members are Gram-negative, non-spore formers, and exhibit an oligotrophic mode of nutrition (Fierer et al., 2005; George et al., 2011; Dedysh and Damsté, 2018). Most of the members are acidophilic chemoheterotrophs growing aerobically under mesophilic conditions (Dedysh and Damsté, 2018). To study this phylum, culture-independent approaches have been more successful rather than culture-dependent approaches. Genomic studies provided insights into the genetic make-over of about ten acidobacterial genomes only (Ward et al., 2009; Kielak et al., 2016a). However, owing to the difficulty in cultivating its members, the in-depth ecological purview of this enigmatic phylum has remained evasive. Since information regarding global distribution patterns and apparent ecological roles of Acidobacteria is inadequate, microbial ecologists are deeply engaged to unfurl this obscure phylum.

## The Requirement for Holistic Approaches for Studying Phylum Acidobacteria

Acidobacteria phylogenetic diversity, richness, abundance, ubiquity, especially in soil ecosystems, pin down their roles in various biogeochemical cycles and broad metabolic versatility (Naether et al., 2012). Although their presence and abundance are confirmed through culture-independent studies but their ecological functions, interrelations with environmental parameters and interactions with other soil microbial communities remain obscure. Significant variations have been encountered during isolation of Acidobacteria strains belonging to different lineages and getting cultivated under specific sets of physicochemical conditions or nearly a narrow range of conditions. This suggests the use of various strategies for the successful recovery of ecologically different Acidobacteria groups.

Acidobacteria diversity and dominance is quite pronounced along with high overall ecological and phylogenetic diversity in contrast to its low cultivation success due to radically different culture laboratory conditions. Although nine different bacterial phyla *viz*., Proteobacteria, Acidobacteria, Actinobacteria, Verrucomicrobia, Bacteroidetes, Chloroflexi, Planctomycetes, Gemmatimonadetes, and Firmicutes are known to dominate in soil (Janssen, 2006; da Rocha et al., 2009), the phylum Acidobacteria represents the most predominant not-yet-cultured, or difficult-to-culture group of bacteria (Janssen, 2006; Foesel et al., 2014), occupying a significant fraction of the soil microbial community. Henceforth, the determination of their ecophysiological roles becomes quite imperative to understand their functional status in complex bacterial communities. Thus, the ecological roles of Acidobacteria have been studied by analyzing the genetic components obtained by complete and/or draft whole-genome sequencing for culturable species and metagenome sequencing for unculturable species.

## 16S rRNA Analyses and Metagenomics in Studying Phylum Acidobacteria

**FIGURE 1:**
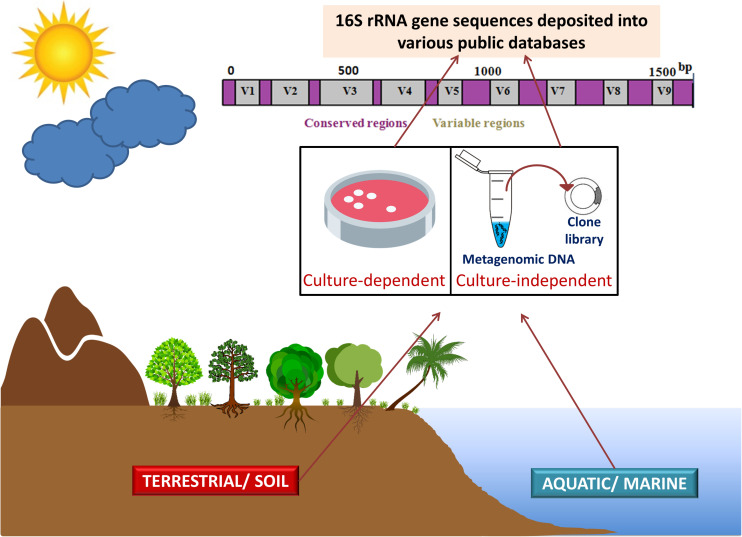
16S rRNA gene sequencing and metagenomics in studying phylum Acidobacteria. Acidobacterial 16S rRNA gene sequences (from culture-dependent studies) and metagenomic DNA information (from culture-independent studies) obtained from various environments (terrestrial ecosystems including soils and aquatic or marine ecosystems) are deposited in various public databases. The amalgamation of 16S rRNA analyses and metagenomics provides deeper insights into the taxonomic and functional diversity of the phylum Acidobacteria. Figure designed using images from pinclipart.com.

Meta-microbiomic (16S rRNA gene-based) studies analyze only one specific gene and not the entire genomes of the community members. The 16S rRNA gene sequences, obtained from various environments by employing culture-dependent and culture-independent approaches, provide deeper insights into the species structure and taxonomic diversity of the phylum Acidobacteria. Studies focusing on 16S rRNA genes provide taxonomic status in any bacterial community, while metagenomics can provide both taxonomic and functional profiles of the microbiota. Metagenomic DNA extracted from the environment is often amplified using group or species-specific primers targeting 16S rRNA genes (Kalam et al., 2017a). Metagenomics has facilitated in an apt understanding of the diversity, abundance, genomic make-up, and ecological roles of acidobacterial members in various ecosystems (Tyson et al., 2004; Venter et al., 2004; Parsley et al., 2011). To obtain a complete community structure, 16S rRNA analyses and metagenomics are often used in conjunction (Figure 1).

During recent years, the sequencing of collective community genomes employing metagenomics has led to a significant breakthrough in understanding the enigmatic phylum Acidobacteria. Subsequent advent and development of several next-generation sequencing (NGS) platforms further enhanced the metagenomic sequencing efficiencies. Metagenomic approaches, however, at few places fail to retrieve genome scaffolds of sizable length due to soil community complexity, intricacy, and absence of genomes (Tringe et al., 2005; Kowalchuk et al., 2007). Recovering genomic information from the soil environment requires the use of large insert strategies (Rondon et al., 2000). Metagenomic DNA being high in molecular weights requires meticulous isolation strategies along with novel cloning and screening methods to facilitate the recovery of large DNA fragments from difficult-to-culture bacterial genomes. Large genomic fragments might contain intact metabolic pathways. Acidobacterial genome fragments were successfully recovered from the environment by using a large metagenomic insert (Liles et al., 2003). Henceforth, such approaches provide important platforms for exploring the hidden biotechnological potential of hitherto uncultured bacteria leading to the discovery of novel organo-chemical compounds (Daniel, 2004).

## Genetic Insights Into the Phylum Acidobacteria

Since the recovery of the first Acidobacteria member and subsequent advances in sequencing technologies has provided a platform to study individual Acidobacteria members at the genomic level. It is quite unfortunate that the number of thoroughly studied genomes of this important phylum is very few, despite their numerical abundance in many environments. Genomic studies have unveiled the hidden physiological and metabolic versatility of Acidobacteria members. Comprehensive studies targeting functional characteristics encoded in acidobacterial genomes may provide new vistas into ecological perspectives of phylum Acidobacteria. Ward et al. (2009) conducted detailed genomic studies for the first time, with three acidobacterial strains [two from subdivision 1 *viz*., *Acidobacterium capsulatum* and *Candidatus* Koribacter versatilis (strain Ellin 345) and one from subdivision 3 *viz*., *Candidatus* Solibacter usitatus (Ellin 6076)]. Further genomic profiling and comparative genomic studies (Männistö et al., 2012; Rawat et al., 2012a; Lee et al., 2015; Eichorst et al., 2018, 2020) provided more profound insights into the acidobacterial genome and ecophysiology.

**FIGURE 2:**
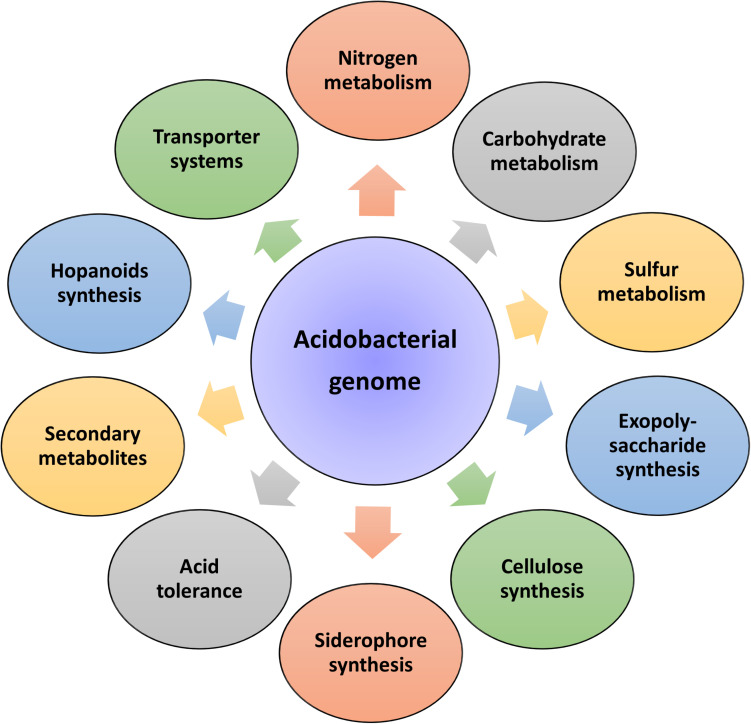
Genetic diversity of the phylum Acidobacteria. Sequencing of acidobacterial genomes and metagenomes revealed a large repertoire of genes responsible for regulating diverse physiological and metabolic functions.

Insights into these genomes reveal large genome size (up to 10 Mbp) and a higher percentage of paralogous genes, which might endow the bacterial strains with potential ecological functions (Challacombe et al., 2011). In the same breath, Acidobacteria genomes also possess a comprehensive physiological set of genes that allows them to adapt to various ecological niches. An overview of the general genomic features of Acidobacteria members and their overall genetic and genomic make-up are respectively represented in Table 1 and Figure 2. Data available from published acidobacterial genome sequences reveal the presence of several genes involved in regulating carbon, nitrogen, and sulfur cycles, and those required for degrading different complex polysaccharides. All cultured Acidobacteria species are reported to produce exopolysaccharide (EPS); the presence of *eps* gene clusters in the genomes further supports this data. The acidobacterial genomes also contain a substantial proportion of genes encoding for various transporters, to tide over stress and starvation, and for the biosynthesis of cellulose, N-acyl-homoserine lactones, polyketides, siderophores, hopanoids, and mobile genetic elements (Ward et al., 2009; Kielak et al., 2016a; Crits-Christoph et al., 2018).

**TABLE 1:** General genomic features of cultured Acidobacteria members with complete genome sequences.

*Acidobacteria species*	*Subdivision*	*Genome size (bp)*	*G* + *C content (mol%)*	*No. of coding sequences*	*Total no. of genes*	*Total protein coding genes*	*Total RNA genes*	*Pseudogenes*	*References*
*Acidobacterium capsulatum* ATCC 51196	1	4,127,496	60.5	3,502	3,425	3,377	48	0	Ward et al., 2009
*Koribacter versatilis* Ellin 345	1	5,650,368	58.4	5,239	4,837	4,779	58	2	Ward et al., 2009
*Granulicella mallensis* MP5ACTX8	1	6,237,577	57.9	NA	4,960	4,907	53	90	Rawat et al., 2012a, 2013
*Granulicella tundricola* MP5ACTX9	1	5,503,984	60.0	NA	4,757	4,705	52	163	Rawat et al., 2012a, 2014
*Terriglobus saanensis* SP1PR4	1	5,095,226	57.3	NA	4,333	4,279	54	99	Rawat et al., 2012a, b
*Terriglobus albidus* ORNL	1	6,405,582	58.5	NA	5,127	5,010	53	64	Podar et al., 2019
*Acidobacterium ailaaui* PMMR2T	1	3,686,523	57.2	NA	3,184	3,131	53	NA	Myers and King, 2016
*Bryocella elongata* DSM 22489	1	5,669,524	62.0	NA	4,620	4,567	53	NA	Pinto et al., 2020
*Acidiphilium rosea* DSM 103428	1	4,213,726	58.8	NA	3,585	3,531	54	NA	Pinto et al., 2020
*Occallatibacter* sp. AB23	1	6,278,575	59.1	NA	5,429	5,367	62	NA	Pinto et al., 2020
*Terracidiphilus gabretensis* S55T	1	5,351,935	57.3	NA	4,610	4,562	48	NA	García-Fraile et al., 2016
*Solibacter usitatus* Ellin 6076	3	9,965,640	61.9	8,568	8,003	7,940	63	114	Ward et al., 2009
*Chloracidobacterium thermophilum*	4	3,695,372	61.3	3,054	NA	NA	NA	NA	Costas et al., 2012
*Chloracidobacterium* sp. CP2_5A	4	3,411,091	64.2	NA	3,083	2,969	55	59	Ward et al., 2017
*Pyrinomonas methylaliphatogenes* K22T	4	3,778,560	59.36	NA	3,244	3,189	55	0	Lee et al., 2015
*Luteitalea pratensis* HEG_-6_39	6	7,480,314	64.7	NA	NA	6,295	NA	NA	Huang et al., 2016
*Holophaga foetida* TMBS4T	8	4,127,237	62.95	NA	3,672	3,615	57	76	Anderson et al., 2012
*Thermoanaerobaculum aquaticum* MP-01T	23	2,660,928	62.7	NA	2,320	2,253	49	18	Losey et al., 2013; Stamps et al., 2014

*NA, not available.*

### Genes for Carbon Metabolism

Each bacterium carries its enzyme machinery catalyzing the breakdown of diverse carbohydrates and nitrogen-containing compounds, which could be used as an identifying characteristic for differentiating varied bacterial species. Acidobacterial genomes possess genes encoding enzymes for the degradation of complex carbohydrate polymers *viz*., xylan, cellulose, hemicelluloses, pectin, starch, and chitin; amino acids, alcohols, and metabolic intermediates (Ward et al., 2009; Belova et al., 2018). In addition to these, gene modules for diverse carbohydrate breakdown, utilization, and biosynthesis within carbohydrate-active enzymes (CAZy) family are also present (Männistö et al., 2012; Rawat et al., 2012a), spanning across 131 glycoside hydrolase (GH) families (Gilbert, 2010). Putative chitinases belonging to GH18 and GH19 family were also identified in the genomes of a few select Acidobacteria (Rawat et al., 2012a). Gene calling and annotation studies of *Acidobacteria* Group 1 *Acidipila* sp. strain EB88 genome indicated that it was rich in glycolytic enzymes and contained about 85 glycoside hydrolases in 48 families (Domeignoz-Horta et al., 2019).

The acidobacterial genomes are flexible and novel in their carbon metabolizing activity. Few select acidobacterial genomes exhibit anaplerotic CO_2_ fixation. Strikingly, homologs of phosphoenolpyruvate carboxylase and isocitrate dehydrogenase have also been detected across several Acidobacteria genomes (Lee et al., 2015; Eichorst et al., 2018). Studies suggest a crucial role of these genes for carbon metabolism in various nutritional pathways as well as a significant role in desiccation resistance, as evidenced by the *Terriglobus saanensis* genome profile (Männistö et al., 2011). A significant contribution is made by acidobacterial enzyme machinery in regulating the carbon biogeochemical cycle (King and Weber, 2007). Since Acidobacteria are endowed with the potential to degrade polymeric carbonaceous complexes, they act as decomposers in soil and actively participate in the cycling of organic matter arising from plants, fungi, and insects (Dedysh and Damsté, 2018).

### Genes for Nitrogen Metabolism

Acidobacteria is well equipped with genes catalyzing the metabolism of inorganic and organic sources of nitrogen (Eichorst et al., 2018). They can effectively reduce nitrate, nitrite, and possibly nitric oxide, as could be evidenced by genomic data, supporting their active participation in nitrogen nutrient circuits. Homolog candidate genes for nitrate reductase (*nirA*) have been identified in Ellin 345, *Geobacter fermentans*, and *Terriglobus aquaticum* (Rajeev et al., 2015), while those for nitrate transport (*nrtABCD*) have also been observed in certain acidobacterial strains. The presence of genes encoding dinitrogenase (*nifD* and *nifK*) and dinitrogenase reductase (*nifH*) have also been reported in one acidobacterial genome (Ward et al., 2009). However, experimental evidence regarding nitrogen fixation by Acidobacteria is missing (Kielak et al., 2016a). Insights into the core genomes provide a wealth of data revealing the presence of putative homologs encoding for extracellular peptidases, which play significant roles in soils by mobilizing ammonium and other intermediates of N-cycle (Bach et al., 2001; Eichorst et al., 2018). Serine endopeptidases are also found to be widely distributed in most Acidobacteria genomes, indicating their proteolytic activity in soil (Brankatschk et al., 2011). Homologs for various extracellular metalloendopeptidases too span across select Acidobacteria genomes, facilitating them for N-uptake during mineral scarcity. Recent studies on the draft genome of *Acidobacteria* Group 1 *Acidipila* sp. strain EB88, isolated from forest soil, indicated that the genome lacked the genes required for organic acid uptake but was equipped with the genes essential for amino acid, ammonium, and nitrate uptake (Domeignoz-Horta et al., 2019).

### Genes for Sulfur Metabolism

*Chloroacidobacterium thermophilium*, a photoheterotrophic member of Acidobacteria subdivision 4, requires reduced sulfur for its growth (Tank and Bryant, 2015). Draft metagenome-assembled genomes of Acidobacteria subdivisions 1 and 3 from peat soil revealed the presence of putative genes for dissimilatory sulfur metabolism (*dsrAB*, *dsrC*, *dsrD*, *dsrN*, *dsrT*, *dsrMKJOP*, *aprBA*, *qmoABC*, *supP*, *hppA*, *sat*) which function under anoxic conditions (Hausmann et al., 2018). The presence of *dsrAB* genes encoding dissimilatory (bi)sulfite reductase suggests the capacity of some Acidobacteria members to perform dissimilatory sulfate/sulfite reduction (Huang et al., 2016; Wasmund et al., 2017; Anantharaman et al., 2018). Certain acidobacterial genomes also encode the *dsrL* gene, which is specific to the sulfur oxidation pathway (Hausmann et al., 2018). The presence of *dsrL* is unusual as it is generally found in sulfur oxidizers rather than in sulfate-reducing microorganisms. However, acidobacterial genomes lack any other genes involved in oxidative sulfur metabolism (Hausmann et al., 2018).

### Genes Encoding Transporters

Different acidobacterial strains harbor an array of genes encoding different ion channels, high-affinity ABC transporters, several other secretory porters, and transport proteins especially required for tiding on oligotrophic conditions (Paulsen et al., 1998; Ward et al., 2009). Genes encoding iron permease FTR1 and FTR2 family proteins and iron transporter genes *viz*., Mn^2+^ and Fe^2+^ transporters are also present in the Acidobacteria genome. The *feoAB* gene encoding a high-affinity ferrous iron transport protein is invariably present in acidobacterial genomes (Velayudhan et al., 2000). The genome of *Terriglobus saanensis* (isolated from the Tundra region) contains abundant genes involved in carbohydrate metabolism and transport (Männistö et al., 2011). Few Acidobacteria genomes also possess multiple copies of genes for siderophore transport *viz*., *tonB, exbB*, and *exbD* (Postle and Kadner, 2003; Llamas and Bitter, 2006; Ward et al., 2009). In addition to these, *pvuE* (candidate gene for vibrioferrin transporter) and *feoB* (candidate gene for enterobactin transporter) are also found in some Acidobacteria genomes (Ward et al., 2009). Acidobacteria members also have genes for the amino acid-polyamine-organocation (APC) superfamily of transport proteins along with dicarboxylate/amino-acid: cation symporter family of secondary transport proteins (Eichorst et al., 2018).

### Genes Regulating Cellulose Synthesis

*Acidobacterium capsulatum* genome contains an operon with a complete set of genes required for cellulose biosynthesis (Ward et al., 2009). Bacterial cellulose is associated with an array of functions facilitating survival in the soil through biofilm formation, retaining moisture under conditions of stress, thus promoting aeration, contributing to soil aggregate formation (Ude et al., 2006; White et al., 2006). Acidobacteria possesses the remarkable capacity to synthesize cellulose, which would promote biofilms to get adhered easily to ferric iron-containing substrates producing biofilm-ferric iron-reducing “bioshrouds” in acidic environments (Johnson et al., 2008). Cellulose biosynthesis via *bcs* operon has been reported in *Terriglobus saanensis* as evidenced through genome sequencing data (Rawat et al., 2012b; de Castro et al., 2013). The genome of *Acidipila* sp. strain EB88 contained genes for biosynthesis and export of capsule and cellulose (Zhang et al., 2018).

### Genes for Oxygen and Hydrogen Utilization

Genomes of particular soil acidobacterial strains possess the potential to respire oxygen at atmospheric and microoxic levels due to the presence of affinity oxidases. Thus, such strains possess an extra selective advantage in the soil microenvironment, where low oxygen concentrations exist (Morris and Schmidt, 2013; Eichorst et al., 2018). The genome of *Acidipila* sp. strain EB88 contains a low-affinity group A heme-copper oxygen oxidase and five high-affinity cbb3 terminal oxidases that enables it to grow under both hypoxic and hyperoxic conditions (Morris and Schmidt, 2013; Domeignoz-Horta et al., 2019). Few Acidobacteria strains possess hydrogen scavenging property due to the presence of nickel-iron [NiFe] hydrogenases (Greening et al., 2015). The occurrence of multiple structural genes (*hhyS*, *hhyL*, *hhyE*) and maturation genes (*hypABCDEF*) required for hydrogenase activity further supports acidobacterial hydrogen utilization (Eichorst et al., 2018).

### Genes Regulating Secondary Metabolite Biosynthesis

Acidobacteria genomes contain biosynthetic gene clusters that encode a vast repertoire of polyketide and non-ribosomal peptide synthases (Crits-Christoph et al., 2018). Additionally, genes regulating the synthesis of diverse secondary metabolites and other natural compounds like siderophores, antifungals, antibiotics, antivirals, antitumor agents, and antinematodal agents have been reported in Acidobacteria genomes (Challis, 2005; Ward et al., 2009; Parsley et al., 2011; Hadjithomas et al., 2015; Crits-Christoph et al., 2018).

### Genes Regulating Stress and Starvation Response

Addiction modules encode various genes, including those required for plasmid maintenance, and has been documented in certain Acidobacteria (Ward et al., 2009). During stress or starvation, the addiction modules operate rapidly and inhibit DNA and protein synthesis (Kroll et al., 2010). This mechanism of stress and starvation tolerance in Acidobacteria enables tiding over environmental oligotrophic nutritional conditions. The prokaryotic transcriptional regulator sigma factor is commonly utilized to control the expression of several gene sets in response to various stresses, including starvation, oxidative stress, heat stress, and exposure to heavy metals that enables the microorganisms to adapt to a stressful environment (Rhodius et al., 2005; Challacombe et al., 2011). The genomes of *Candidatus* Solibacter usitatus [Ellin 6076] and *Candidatus* Koribacter versatilis [Ellin 345] contain a vast repertoire of sigma E (σ^E^) homologs (70 and 28 homologs, respectively) that are induced during starvation and other stress conditions (Challacombe et al., 2011).

The presence of hydrogenases enables specific acidobacterial strains to consume atmospheric hydrogen (Greening et al., 2015), which could be a strategy to overcome starvation (Eichorst et al., 2018). Certain Acidobacteria members are equipped with the genes responsible for the dissimilatory reduction of nitrite to ammonia (*nrfHA*), which not only provides energy supply but also aids in the detoxification of nitrosative stress (Rajeev et al., 2015; Eichorst et al., 2018). A very recent study by Pinto et al. (2020) reported that carotenoid production by *Occallatibacter* sp. (belonging to Acidobacteria subdivision 1) can confer tolerance to environmental oxidative stress. Thus, the production of carotenoids and related compounds may offer competitive benefits to soil Acidobacteria.

### Genes Regulating Acid Tolerance

Most Acidobacteria members prefer acidic conditions (3.0–6.5 pH) for their growth (Sait et al., 2006; Ward et al., 2009). Also, lower pH levels support a higher abundance of Acidobacteria (Männistö et al., 2007; Lladó et al., 2018). Microorganisms equipped with acid resistance (AR) systems are likely to survive in highly acidic conditions (Sun et al., 2012). Certain moderately acidophilic acidobacterial strains *viz*., *A*. *capsulatum*, Ellin 345 (*K*. *versatilis*), and Ellin 6076 (*S*. *usitatus*) contain candidate genes in the AR3 (arginine-dependent AR) system indicating the presence of an acid tolerance system (Ward et al., 2009; Kielak et al., 2016a). However, the strains lack the genes involved in other inducible AR systems, *viz*., AR1 (oxidative AR), AR2 (glutamate-dependent AR), and AR4 (lysine-dependent AR).

### Genes for Synthesis of Hopanoids

Hopanoids are pentacyclic triterpenoid bacterial membrane lipids facilitating cell membrane permeability, especially during extreme environmental conditions (Damsté et al., 2017). Genes related to hopanoid biosynthesis (*shc* gene) have been detected in *Cadidatus* “K. versatilis” belonging to subdivision 1 isolated from soil (Joseph et al., 2003). The presence of C_30_ hopanoids and bacteriohopane polyols was reported in multiple Acidobacteria subdivisions, evident from the occurrence of respective biosynthesis genes (*hpnA*, *hpnG*, *hpnH*) in acidobacterial genomes (Damsté et al., 2017).

### Genes for Exopolysaccharide Biosynthesis

Genomic mining studies suggest that Acidobacteria belonging to subdivision 1 can encode EPS biosynthesis genes (Ward et al., 2009). Additionally, several cultured Acidobacteria species are known to secrete EPS (Eichorst et al., 2007; Pankratov and Dedysh, 2010; Whang et al., 2014; Kielak et al., 2017). Gene prediction, along with functional annotation data, identifies a gene cluster encoding capsular polysaccharide in the genomes of three acidobacterial strains isolated from tundra soil. *Granulicella mallensis* genome possessed the *epsH* gene involved in exopolysaccharide synthesis (Rawat et al., 2012a).

### Genes for Acyl-Homoserine Lactones (AHL)

Quorum sensing molecules, like acyl-homoserine lactones (AHL), aid in coordinating gene expression in bacterial populations (Parsek et al., 1999). Genomes of Acidobacteria contain a considerable fraction of genes encoding for biosynthesis of N-acyl homoserine lactones (Ward et al., 2009).

### Genes for Mobile Genetic Elements

Mobile genetic elements like transposons, bacteriophages, insertion sequence (IS) elements, and integrative and conjugative elements (ICEs) present in several Acidobacteria are known to confer shape and plasticity to the acidobacterial genome. These elements are speculated to mediate horizontal gene transfer, aid in the evolution and ecological success of Acidobacteria (Frost et al., 2005). A recent meta-study by Eichorst et al. (2018) identified 35 putative prophages across 19 acidobacterial genomes. Insertion sequence families encoding their mobility patterns also have been reported to be spanning across Acidobacteria genomes (Challacombe and Kuske, 2012). Multiple genes encoding mobile elements, including transposases and IS elements, have been identified in the genome of *Candidatus* “Solibacter usitatus” Ellin 6076. Additionally, the genome also harbors genes encoding phage integrase family proteins and several other proteins containing a retroviral integrase catalytic region domain, catalyzing site-specific recombinations (Challacombe and Kuske, 2012).

## Excavating the Ecological Roles of Phylum Acidobacteria in Soil

**FIGURE 3:**
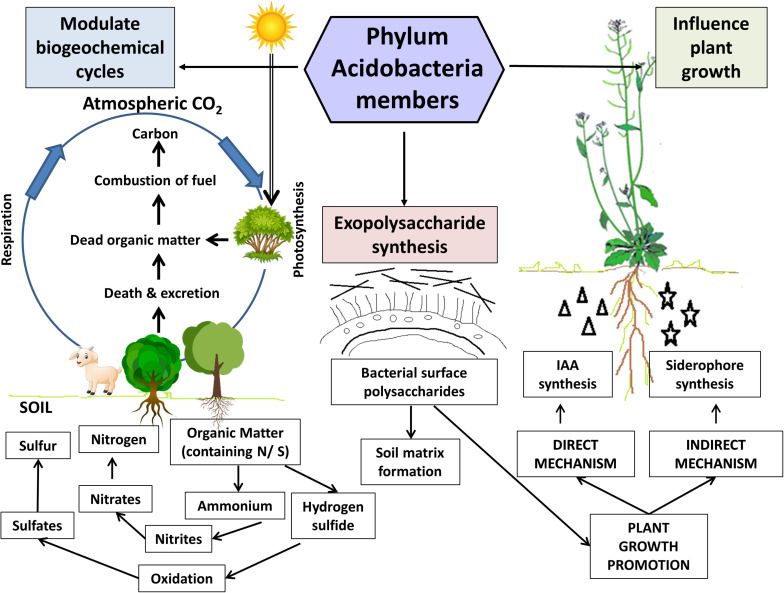
Ecological roles of Acidobacteria members in the soil. Acidobacteria members in the plant-soil ecosystem facilitate modulation of critical biogeochemical cycles *viz*., carbon, nitrogen, and sulfur cycles. Exopolysaccharides (produced by almost all cultured members) aid in soil matrix formation and contribute to plant growth promotion by facilitating water and nutrient uptake by plants. The representatives belonging to *Granulicella* and *Acidocapsa* genera exhibit *in vitro* plant growth promotion traits in the model plant *Arabidopsis thaliana*. These acidobacterial strains effectively produced the phytohormone indole-3-acetic acid (IAA) and siderophores, which significantly enhanced the plant growth parameters (shoot and root biomass). Figure designed using images from pinclipart.com.

Despite the recent progress in the field of acidobacterial ecology, there is still a paucity of complete information regarding their ecophysiological roles. Significant ecological functions have been reported in forest soil for Acidobacteria members (García-Fraile et al., 2016; Liu et al., 2016). Acidobacteria members in plant-soil ecosystems play pivotal ecological roles, including modulation of biogeochemical cycles and influencing plant growth. The key findings from relevant studies on soil Acidobacteria, highlighting their salient genomic features and ecological roles, are summarized in Table 2. An overview of their ecological roles in the plant-soil ecosystems is diagrammatically represented in Figure 3 and is discussed below.

**TABLE 2:** Salient genomic features and ecological roles of soil Acidobacteria.

Salient features	References
Involvement in carbon cycle	King and Weber, 2007; Banerjee et al., 2016; García-Fraile et al., 2016; Belova et al., 2018; Dedysh and Damsté, 2018
Involvement in nitrogen cycle	Ward et al., 2009; Rajeev et al., 2015; Eichorst et al., 2018
Involvement in sulfur cycle	Wasmund et al., 2017; Hausmann et al., 2018
Involvement in plant growth promotion	Kielak et al., 2016b; Kalam et al., 2017a
Involvement as “keystone taxa”	Banerjee et al., 2016; Jiang et al., 2017; Li et al., 2017; Banerjee et al., 2018
Involvement in soil matrix formation	Kielak et al., 2016a; Kielak et al., 2017
Establishment of biofilms	Ward et al., 2009; Kielak et al., 2016a; Kielak et al., 2016b
Production of exopolysaccharides	Ward et al., 2009; Rawat et al., 2012a; Kielak et al., 2017
Biosynthesis of secondary metabolites	Ward et al., 2009; Parsley et al., 2011; Hadjithomas et al., 2015; Damsté et al., 2017; Crits-Christoph et al., 2018
Tolerance to stress, starvation, and acidity	Ward et al., 2009; Challacombe et al., 2011; Morris and Schmidt, 2013; Greening et al., 2015; Rajeev et al., 2015; Eichorst et al., 2018; Domeignoz-Horta et al., 2019; Pinto et al., 2020
Presence of mobile genetic elements	Frost et al., 2005; Challacombe and Kuske, 2012; Eichorst et al., 2018

### Acidobacteria as “Keystone Taxa” in Soil Ecosystems

A recent study by Banerjee et al. (2018) has reviewed and re-defined “keystone taxa” in microbial ecology. Microbial keystone taxa have been often ascribed as “ecosystem engineers” as they are the unequivocal drivers of microbial community structure and function in different ecosystems. Acidobacteria was reported to be one of the keystone bacterial taxa in soil associated with the decomposition of soil organic matter (SOM), implying their significance in carbon turnover (Banerjee et al., 2016). Computational inferences obtained from analyses of various terrestrial ecosystems and habitats indicate several subdivisions of phylum Acidobacteria represent the keystone taxa in grasslands (subdivision 4), forest or woodlands (subdivision 4), agricultural soils (subdivision 17), and plant-associated microbiota (subdivisions 1, 3, and 6) (Banerjee et al., 2016, 2018; Jiang et al., 2017; Li et al., 2017). Speculations suggest that these microbial residents exert beneficial effects by selectively modulating the ecological processes of host ecosystems. These bacteria offer two crucial ecological services *viz*., SOM decomposition, and denitrification, thereby enhancing carbon stability (Banerjee et al., 2018). Successful manipulation of these soil dwellers might augment the key ecological process of soil carbon sequestration. Since Acidobacteria members have been identified as structural and functional keystones in plant-soil microbiomes and agroecosystems, they can be exploited to enhance crop performance and productivity.

### Modulation of Biogeochemical Cycles

The gamut of living organisms invariably depends on the supply of essential elements such as oxygen, hydrogen, carbon, nitrogen, phosphorus, and sulfur for growth and survival. In any ecosystem, albeit biogeochemical cycles or nutrient cycles are regulated by the biotic and abiotic components of that system, but microorganisms play a key role in modulating them (Falkowski et al., 2008). The critical metabolic processes *viz.*, nitrogen metabolism, carbon fixation, and methane metabolism, sulfur metabolism operating in microbes effectively control global biogeochemical cycling.

Genomic studies highlight the carbohydrate utilization potential exhibited by Acidobacteria members (Ward et al., 2009; Kielak et al., 2016a). The presence of genes encoding enzymes for the degradation of complex carbohydrate polymers like cellulose, hemicellulose, chitin, xylan, and lignin derivatives signifies their active participation in the carbon circuit as decomposers in soil (Ward et al., 2009; Wegner and Liesack, 2017; Banerjee et al., 2018; Belova et al., 2018) and cycling of organic matter derived from plants, fungi, and insects (King and Weber, 2007; García-Fraile et al., 2016; Dedysh and Damsté, 2018).

Soil bacteria convert the inorganic nitrogen to organic compounds (Canfield et al., 2010), which are, in turn, utilized by the plants and other microbes. In plant-soil ecosystems, Acidobacteria communities represent an important microbial guild, central in nitrogen cycling. Genomic evidence coerces the crucial role of Acidobacteria in N-cycling in soils (Ward et al., 2009). Specialized microorganisms metabolize diverse sulfur compounds via redox reactions and contribute to driving the global sulfur cycle (Wasmund et al., 2017). Acidobacteria genome harbors genes regulating sulfur metabolism that were found to be expressed in native peat soils and upregulated in diverse anoxic conditions, indicating their active role in modulation of the environmental sulfur cycle (Hausmann et al., 2018). Hausmann et al. (2016) reported that consortia of diverse sulfate-reducing microorganisms, including Acidobacteria members, drive the sulfur reduction in peatlands and Acidobacteria members (*Holophaga*) responded positively to sulfate stimulation in peat soil microcosms.

### Exopolysaccharide Production

Exopolysaccharides are bacterial polysaccharides synthesized extracellularly (Nwodo et al., 2012), which play a critical role in the soil matrix and aggregate formation (Bogino et al., 2013). EPS plays a significant role in the development of mature biofilm by acting as bridges between cell surfaces (Bura et al., 1998). In the rhizosphere zone, EPS production by bacterial populations contributes toward nutrient and water uptake by plant roots through modification of the physicochemical properties of rhizosphere soil (Degens et al., 1994; Kalam et al., 2017b) and aids in establishing interactions with the root appendages for successful plant-microbe interactions (Bianciotto et al., 2001, 2009).

Several cultured Acidobacteria species are known to secrete EPS (Eichorst et al., 2007; Kielak et al., 2017). Genomic analyses indicate that Acidobacteria belonging to subdivision 1 can encode EPS biosynthesis genes (Ward et al., 2009). These EPSs might protect Acidobacteria, endowing them the ability to survive for prolonged periods in soil (Kielak et al., 2017). Recently, the characterization of EPSs derived from two subdivisions 1 acidobacterial strain (*Granulicella* sp.) has indicated them to be possessing potential environmental, bioremediation, and biotechnological applications (Kielak et al., 2017). These EPSs could be used as natural eco-friendly binders and formulants in the biofertilizer industry.

Acidobacterial genome profiling validates the presence of cellulose synthesis genes along with related accessory proteins. Studies postulate several Acidobacteria members to possess the capability to establish biofilms, exhibit resistance toward desiccation, and aid in the formation of soil aggregates (Kielak et al., 2016a). However, physiological data confirming actual EPS production by Acidobacteria and validating their ecophysiological roles remains obscure.

### Acidobacteria as Plant Growth Promoting Rhizobacteria (PGPR)

Plant root microbiome harbors beneficial microbes, which offer an eco-friendly alternative to improve plant growth and protect against phytopathogens (Bulgarelli et al., 2013; Philippot et al., 2013). Plant growth promoting rhizobacteria (PGPR) have been defined as free-living soil bacteria dwelling in the rhizosphere and endowed with the potential to stimulate plant growth and crop yield (Dutta and Podile, 2010; Sayyed et al., 2019; Zope et al., 2019). A plethora of reviews exhaustively documents almost all aspects of PGPR (Ahmad et al., 2008; Lugtenberg and Kamilova, 2009; Etesami et al., 2015; Parray et al., 2016; Backer et al., 2018). PGPR, through their direct and indirect effects, can bring about substantial plant growth promotion (PGP). They act directly by facilitating nitrogen absorption and assimilation, mineral solubilization, production of phytohormones (Ahmad et al., 2008; Parray et al., 2016; Shaikh et al., 2018; Kalam et al., 2020). Indirectly, PGPR, through their biocontrol mechanisms, produce siderophores (Wani et al., 2016; Sayyed et al., 2019), lytic enzymes (Jadhav et al., 2020a, b), antibiotics (Vinay et al., 2016; Reshma et al., 2018; Kenawy et al., 2019), 1-aminocyclopropane-1-carboxylic acid (ACC) (Glick, 2014; Goswami et al., 2016; Sagar et al., 2020), guarding host plants against pathogens (Shaikh et al., 2018).

Several studies reported Acidobacteria to be avid rhizosphere colonizers (Lee et al., 2008; da Rocha et al., 2010, 2013). The first evidence for PGP by Acidobacteria subdivision 1 members was provided by Kielak et al. (2016b). Their experiments confirmed interactions between three acidobacterial strains belonging to the genera *Granulicella* and *Acidicapsa* and the host plant *Arabidopsis thaliana*. Determination of *in vitro* PGP traits provided evidence that all the test strains were active producers of phytohormones IAA and siderophore. There was a significant increase in root growth parameters, although shoot biomass variations were non-significant in comparison to the controls. All three strains were able to adhere to roots, form a biofilm, and grow along the root surface. The pioneering study provided the first time with a direct confirmation regarding the Acidobacteria*-*plant interactions and PGP by Acidobacteria members. Acidobacteria subdivision 1 member dominates among other subdivisions, followed by subdivisions 4, 3, 8, and 23. Their preponderance in the soil environment in comparison with other subdivisions and their genetic profiles surmise their active roles in plant-soil ecosystems. Also, analysis of biosynthetic gene pathways from few publicly available genomes of soil-inhabiting acidobacterial isolates suggests the presence of genes linked to the production of secondary metabolites involved in plant growth (Parsley et al., 2011; Hadjithomas et al., 2015).

The diversity and composition analysis of broomcorn millet rhizosphere using 16S rDNA sequencing indicated Acidobacteria to be a core bacterial component among rhizobacterial assemblages, comprising 10.7% of the total operational taxonomic units (OTUs) obtained (Na et al., 2019). In another interesting study, Kalam et al. (2017a) explored the effects of three PGPR strains *viz.*, *Sphingobacterium* sp., *Variovorax* sp., and *Roseomonas* sp. on crop rhizospheric population densities of acidobacterial members. They reported that the equivalent cell numbers of Acidobacteria members gradually increased over time with a simultaneous increase in plant growth promotion by PGPR inocula. The study speculated the existence of beneficial interactions between the triad of difficult-to-culture bacteria, PGPR, and host plants. Additional experiments in plant-soil ecosystems are still required for unveiling the exact ecological roles of rhizospheric Acidobacteria members.

## Conclusion

Soil represents a luxurious pool of microorganisms, including the shadow biosphere dwellers, the difficult-to-culture bacteria which might be strategic sources for novel and successful biotechnological products. Acidobacteria is an important and among the most abundant difficult-to-culture phylum harboring the soil ecosystem. Acidobacteria plays significant ecological roles, as evidenced through their active participation in key carbon, nitrogen, and sulfur biogeochemical circuits. At the same time, the production of EPS further strengthens their ecophysiological functions in establishing plant-soil beneficial interactions. Since this divergent phylum predominates rhizosphere microbial communities, these bacteria might be a significant component of the crop’s natural environment. Initiatives for manipulating crop rhizosphere with acidobacterial populations to increase plant growth in laboratory and greenhouse studies could be considered to be future challenges and thrust areas for research. The correlation and information presented in this review might prove to be relevant for future modeling of experiments to expose the potential biotechnological roles of Acidobacteria members and exploit them as prospective alternatives to agrochemicals, facilitating sustainable agriculture.

## Author Contributions

SK and AB contributed equally to the conception and design of the review and wrote the first draft of the manuscript. IA and RS critically reviewed and edited the manuscript. HE-E, DD, and NS wrote portions of the manuscript. All authors read and approved the final version of the manuscript.

## Conflict of Interest

The authors declare that the research was conducted in the absence of any commercial or financial relationships that could be construed as a potential conflict of interest.
